# A Herbivorous Mite Down-Regulates Plant Defence and Produces Web to Exclude Competitors

**DOI:** 10.1371/journal.pone.0023757

**Published:** 2011-08-24

**Authors:** Renato A. Sarmento, Felipe Lemos, Cleide R. Dias, Wagner T. Kikuchi, Jean C. P. Rodrigues, Angelo Pallini, Maurice W. Sabelis, Arne Janssen

**Affiliations:** 1 Graduate Programme in Plant Science, Federal University of Tocantins (UFT), Gurupi, Tocantins, Brazil; 2 Section Population Biology, Institute for Biodiversity and Ecosystem Dynamics, University of Amsterdam, Amsterdam, The Netherlands; 3 Department of Entomology, Federal University of Viçosa, Viçosa, Brazil; Institut Mediterrani d'Estudis Avançats (CSIC/UIB), Spain

## Abstract

Herbivores may interact with each other through resource competition, but also through their impact on plant defence. We recently found that the spider mite *Tetranychus evansi* down-regulates plant defences in tomato plants, resulting in higher rates of oviposition and population growth on previously attacked than on unattacked leaves. The danger of such down-regulation is that attacked plants could become a more profitable resource for heterospecific competitors, such as the two-spotted spider mite *Tetranychus urticae*. Indeed, *T. urticae* had an almost 2-fold higher rate of oviposition on leaf discs on which *T. evansi* had fed previously. In contrast, induction of direct plant defences by *T. urticae* resulted in decreased oviposition by *T. evansi*. Hence, both herbivores affect each other through induced plant responses. However, when populations of *T. evansi* and *T. urticae* competed on the same plants, populations of the latter invariably went extinct, whereas *T. evansi* was not significantly affected by the presence of its competitor. This suggests that *T. evansi* can somehow prevent its competitor from benefiting from the down-regulated plant defence, perhaps by covering it with a profuse web. Indeed, we found that *T. urticae* had difficulties reaching the leaf surface to feed when the leaf was covered with web produced by *T. evansi*. Furthermore, *T. evansi* produced more web when exposed to damage or other cues associated with *T. urticae*. We suggest that the silken web produced by *T. evansi* serves to prevent competitors from profiting from down-regulated plant defences.

## Introduction

Interspecific competition occurs when adding individuals of one species reduces the population size of another species that shares the same resource [1], [2]. Herbivores may directly affect individuals of other species that share the same host plant through interference competition and indirectly by reducing the food quantity of the shared host (exploitative competition). Herbivores competing for plants may also affect individuals of the other species indirectly by altering the quality of the shared host plant [3] through induction of changes in the primary and secondary chemistry of the shared host plant, which in turn affects the competitor's performance [4], [5], [6], [7]. For instance, herbivores are known to induce plant defences [5], [8], [9], [10], [11], and these plant defences may negatively affect the performance of other herbivores on the same plant [3], [12]. The induction of such plant defences and the tolerance to them by a herbivore species may determine the outcome of its competition with other, less tolerant herbivores. Alternatively, plants can also become more susceptible to attacks by herbivores after previous attacks by other species of herbivores [5]. In these cases, negative cross-talk between defensive pathways may be involved [13].

In this article, we report on the interactions between the herbivorous spider mite *Tetranychus evansi* Baker & Pritchard (Acari: Tetranychidae) and *Tetranychus urticae* Koch (Acari: Tetranychidae) on tomato plants. It is well known that *T. urticae* induces plant defences resulting in reduced performance on previously attacked plants [3], including tomato [10], [14], [15]. We recently found that *T. evansi* down-regulates plant defences in the leaves of tomato plants, resulting in an increased performance of *T. evansi* on plants attacked by conspecifics [16]. Hence, this is exactly the opposite pattern as for *T. urticae* and many other species [17], [18], [19]. The danger of such down-regulation is that plants could also become a more profitable resource for heterospecific competitors such as *T. urticae*. We investigated whether this competing species could indeed profit from the changes in tomato plants induced by *T. evansi*, and how *T. evansi* can prevent its competitors from profiting.

In many parts of the world, both *T. evansi* and *T. urticae* are important pests of solanaceous crops, and attack tomato plants (*Solanum lycopersicum*), on which they can potentially compete [20], [21], [22]. Many spider mites produce a silken web over the leaves they feed on and the offspring they produce [23]. This web is hypothesized to serve as a defence against predators, and indeed, many predator species are incapable of penetrating the dense web produced by some spider mite species [24]. However, it is possible that the web also affects interspecific interactions among herbivore species, i.e. that it also offers protection against competitors [25].

There are large interspecific differences in the amount of silk produced and in the structure of the web of spider mite species [23], [26], [27], [28]. Both *T. urticae* and *T. evansi* produce copious amounts of web, but especially the latter is known for its extreme web production [22], [29]. We investigate here, whether this profuse web may serve to protect the plant parts with reduced defences from competitors. Specifically, we addressed the following questions: (1) Can *T. urticae* profit from the reduced direct defence of plants attacked by *T. evansi* and is *T. evansi* sensitive to the defences induced by *T. urticae*? (2) Are the two spider mites affected by each others' web? (3) What is the effect of these interactions on the outcome of competition between the two species on tomato plants? (4) Does the presence of competitors stimulate *T. evansi* to produce more web?

## Materials and Methods

### Mite rearing and plant material

*Tetranychus evansi* was obtained in 2002 from a population infesting tomato plants (*Solanum lycopersicum*, variety Santa Clara I-5300) in a greenhouse at the Federal University of Viçosa, Brazil. The population of *T. urticae* was obtained from infested tomato plants of the same variety as described above in a greenhouse at the Federal University of Viçosa. Both *T. evansi* and *T. urticae* were cultured on tomato plants (variety Santa Clara I-5300). Because the rate of oviposition varies with age in spider mites [28], young adult females of *T. urticae* and *T. evansi* of similar age were used in all the experiments. To obtain such cohorts, several adult females were allowed to lay eggs on detached uninfested tomato leaves on wet cotton wool, placed inside a tray. The adults were removed after 24 h and the mites hatching from the eggs were reared until adulthood.

Tomato seeds were sown in trays with a commercial substrate (Bioplant®, Bioplant Misturadora Agrícola LTDA, Nova Ponte, MG), composed of vermiculite and organic fertilizer and were kept in mite-proof screen cages in a greenhouse. Plants (21 days old) were transplanted to plastic pots (2L) that contained a mixture of soil, bovine manure (3∶1) and fertilizer (4-14-8 N-P-K). The plants were subsequently grown in mite-proof screen cages in a greenhouse until they were 45 days old and had at least four completely developed leaves. Subsequently, the plants were used either for the experiments or for spider-mite rearing.

### Spider mite oviposition

To evaluate the effect of damage and web of *T. evansi* on the oviposition of *T. urticae*, discs (diam. = 1 cm) were made from leaves of clean tomato plants and subsequently infested with *T. evansi* females. The leaf discs were placed in groups of 10 in trays (20×10 cm) containing a piece of foam rubber covered with wet cotton wool and soaked with water to prevent the mites from escaping from the discs. Each disc received 10 mated *T. evansi* females, which were allowed to produce web and feed for a period of two days. Thereafter, all the females were carefully removed from the leaf discs, while taking care not to damage the web [30]. This resulted in discs that were damaged by feeding activities of *T. evansi* and entirely covered with a fine layer of web. The eggs and web were removed from about half of these discs, resulting in discs with damage by *T. evansi*. The eggs and web were left intact on the other half of the discs, and the eggs were counted. As a control, leaf discs from clean plants, without damage and web of *T. evansi*, were used. Subsequently, one 2-day-old mated female *T. urticae* was released on each disc, and the discs were incubated inside a climate box (28±2°C; relative humidity 70±10% and 12 hours of light) for a period of four days. Subsequently, the eggs produced by the *T. urticae* females were counted. Each replicate consisted of a group of 10 leaf discs and each treatment was replicated 11 times through time.

Likewise, we performed the reverse experiment, evaluating the effect of damage and web of *T. urticae* on oviposition by *T. evansi*. Leaf discs were treated as above, but with *T. urticae* instead of *T. evansi*, and the production of eggs by 2-day-old mated female *T. evansi* was measured on leaf discs that were previously damaged by *T. urticae* and contained eggs and web of this species, leaf discs that were previously damaged by *T. urticae*, but without eggs and web, and clean leaf discs. The rate of oviposition of *T. urticae* and *T. evansi* in each experiment was analysed using a linear mixed-effects models (lme) with treatment as fixed effect and replicate as a random factor (see Statistical Tables S1). Contrasts among treatments were calculated by aggregating non-significant treatment levels in an *a posteriori* stepwise procedure [31]. All analyses were done using R statistical software [32].

### Competition on plants

Tomato plants were infested with three 2-day-old mated females of *T. evansi*, *T. urticae*, or both, using a fine brush. There were three treatments, each replicated four times (i.e. four plants): plants infested with either species separately and plants infested with both species. When plants were simultaneously infested with both species, the two species were placed on different leaves. Hence, if *T. evansi* would down-regulate plant defence throughout the plant, we would expect *T. urticae* to profit from it and perform better on plants with *T. evansi* than if alone. *Tetranychus urticae* systemically induces direct plant defences, such as the increased activity of proteinase inhibitors [14], [16], and these defences may negatively affect *T. evansi*. Plants were maintained inside a climate room (28±2°C; relative humidity 70±10% and 12 hours of light) during the entire experimental period. On the plants, adult female mites of each of the two mite species were counted every 3 days until the plants died of overexploitation by the mites (after 33–35 days). The two species could easily be distinguished because adult females of *T. evansi* are red and the strain of *T. urticae* used here is white with two clearly visible dark spots. Log-transformed densities of spider mites were analyzed using linear mixed-effects models (lme), to correct for repeated measures with the presence/absence of the other species and time as factors and replicate as random factor.

### Does web of *T. evansi* hinder *T. urticae*?

In this experiment, we tested whether *T. urticae* was hindered by the web of *T. evansi*. We assessed whether the mites were capable of reaching the leaf surface when it was covered with web of *T. evansi*. *Tetranychus urticae* usually resides on the surface at the underside of leaves, where they spend 50% of their time feeding [33]. Leaflets of clean tomato plants were put upside down on moist filter paper, which in turn was put on a sponge inside a Petri dish (diam. 13.5 cm) filled with water. The edges and the petiole of the leaflet were covered with moist cotton wool to prevent mites from escaping and to maintain leaf turgor. One hundred adult female *T. evansi* were put on each leaflet and were incubated for 4 days (25±5C; 70±10%, 12∶12 L∶D photoperiod) to produce web. Subsequently, the web was gently removed from half of the leaflets with a fine brush. This treatment did not result in complete removal of the web, and occasionally one or a few mites were removed together with the web (0–15 per leaflet). A small strip of Parafilm® (3×6 mm) was put on top of the central vein, and 10 adult female *T. urticae* were released on this strip, from where they could descent onto the web or the leaf. Their position (on the strip, in the web, on the leaf surface or dead/escaped) was evaluated 1.5 h and 17.5 h after release. Moreover, we assessed which mites were feeding, judged by an inclined posture and the mouthparts (pedipalps) touching the leaf, after 17.5 h. In case of doubt, we checked for movements of fluid through the mite gut, which is a genuine sign of feeding. Ten replicates (leaflets) were done per treatment (with web removed or left intact). The data were analysed with a generalized linear model (glm) with a binomial error distribution and the presence or absence of web as fixed effect; mites that had died or escaped were omitted from the analysis.

### Webbing and oviposition by *T. evansi*

In this experiment, we assessed the effect of cues associated with *T. urticae* on oviposition and web production by *T. evansi*. Prior to measuring the oviposition and webbing of *T. evansi*, plants were infested with either *T. evansi* or *T. urticae* by putting four small, infested tomato leaflets on each plant, inside a mite-proof screen cage in a greenhouse. As a control, a group of plants of the same age was left clean. The plants were incubated in this way for one week, resulting in 300–400 adult mites per plant with mites. Subsequently, the web, mites and eggs were removed from leaflets using a fine brush, and five leaf discs (Ø = 1 cm) were punched from the leaflets of each plant. Seven replicates were done in blocks through time, with one plant for each treatment per block. The leaf discs were kept in groups of 15 in plastic trays (30×20 cm) containing a piece of foam rubber covered with wet cotton wool and filled with water to prevent the mites from escaping from the discs. One 2-day-old mated female *T. evansi* from a cohort was individually placed on each leaf disc. Four days later, the eggs produced by the mites were counted and the web produced was measured with a technique adapted from [34] [see 35]. Soil particles (0.177 mm) were sprinkled over a leaf disc with web, using a fine brush. Particles were counted and their position (in the web or on the leaf) scored using a stereo microscope (32× magnification). Web density was calculated as the percentage of particles that was found suspended in the web [35].

Rates of oviposition rates and web densities were averaged per plant and these averages were analysed with linear mixed effects models (lme) with treatment (damage by *T. evansi*, by *T. urticae* or no damage) as fixed effect and replicate as random factor. Contrasts among treatments were calculated as above.

### Effect of conspecific and heterospecific cues on oviposition and web production by *T. evansi*

Here, we evaluated the effect of cues emanating from leaves infested with *T. evansi* or *T. urticae*, as well as from clean leaves (control) on the production of eggs and web by *T. evansi* females. A tomato leaflet infested with 50 *T. evansi* or 50 *T. urticae* females or a clean leaflet was placed in the centre of a Petri dish (diam. = 10 cm) containing wet cotton wool and this leaflet served as a source for cues. Subsequently, a group of 6 clean leaf discs (Ø = 1 cm) were placed in a circle around the leaflet, and a mated *T. evansi* female, 2-day-old since her last moult, was placed on each leaf disc. Thus, these leaf discs received volatile cues as well as water-soluble cues from the leaflet in the centre. Each replicate consisted of a group of 6 leaf discs in a Petri dish. Each Petri dish (Ø = 10 cm) was placed inside a plastic tray that was sealed with PVC film and kept inside a climate room (28±2°C; relative humidity 70±10% and 12 hours of light). Each treatment was replicated 23 times (number of Petri dishes).

Because not all experiments were carried out simultaneously, and plants may differ with season and season-specific greenhouse conditions, we carried out the experiment in blocks through time, each block consisting of an equal number of each treatment. The results of the different treatments should therefore be compared to their respective controls, carried out at the same time. The oviposition rate and relative web density were analysed using linear mixed-effects models (lme) with replicate as random factor. Contrasts among treatments were calculated as above.

## Results

### Spider mite oviposition

**Figure 1:**
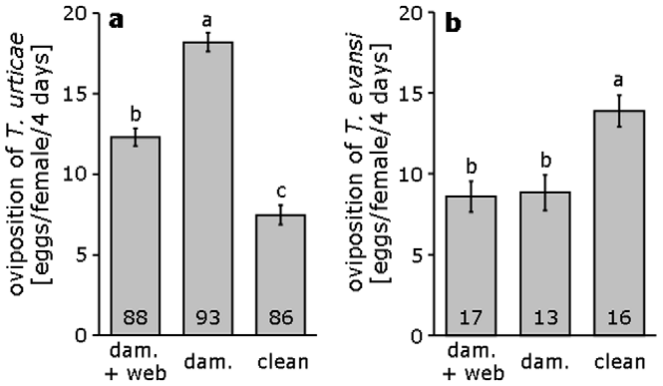
Down-regulation of plant defence by *T. evansi*. **a** Mean (+s.e.m.) oviposition rate of *T. urticae* on tomato leaf discs with damage and web of *T. evansi* (dam.+web), on leaf discs with damage by *T. evansi*, but without web (dam.), and on clean leaf discs (clean). **b** Mean (+s.e.m.) oviposition rate of *T. evansi* on tomato leaf discs with damage and web of *T. urticae*, on leaf discs with damage but without web of *T. urticae*, and on clean leaf discs. Different letters denote significant differences among treatments (lme with log likelihood ratio test). Experiments of panels a and b were not carried out at the same time for logistical reasons. Treatments should therefore be compared to the control carried out at the same time, presented in the same panel. Numbers inside the bars give number of replicates.

There was a significant effect of treatments on the rate of oviposition of *T. urticae* (lme, Log likelihood ratio (L-ratio) = 185.8, d.f. = 2, *p*<0.0001, Fig. 1a); the oviposition of *T. urticae* was highest on leaf discs with damage but without web of *T. evansi*, intermediate on leaf discs with damage and webbing of *T. evansi*, and lowest on clean leaf discs (Fig. 1a, Log likelihood ratio (L-ratio), all *p*'s<0.0001). The rate of oviposition of *T. evansi* was also affected by treatment (lme, L-ratio = 16.7, d.f. = 2, *p* = 0.0002). It was significantly higher on clean leaf discs than on discs previously damaged by *T. urticae*, either with or without web (Fig. 1b, L-ratio = 14.2 and 11.4 respectively, *p*'s<0.001). There was no effect of the presence of web produced by *T. urticae* on oviposition of *T. evansi* (L-ratio = 0.043, *p* = 0.84). These results show that *T. urticae* can profit from the reduced direct defence of plants attacked by *T. evansi*, but was negatively affected by *T. evansi* web. Furthermore, *T. evansi* was not hindered by the web of *T. urticae*, but was negatively affected by the defence induced by *T. urticae*.

### Competition on plants

**Figure 2:**
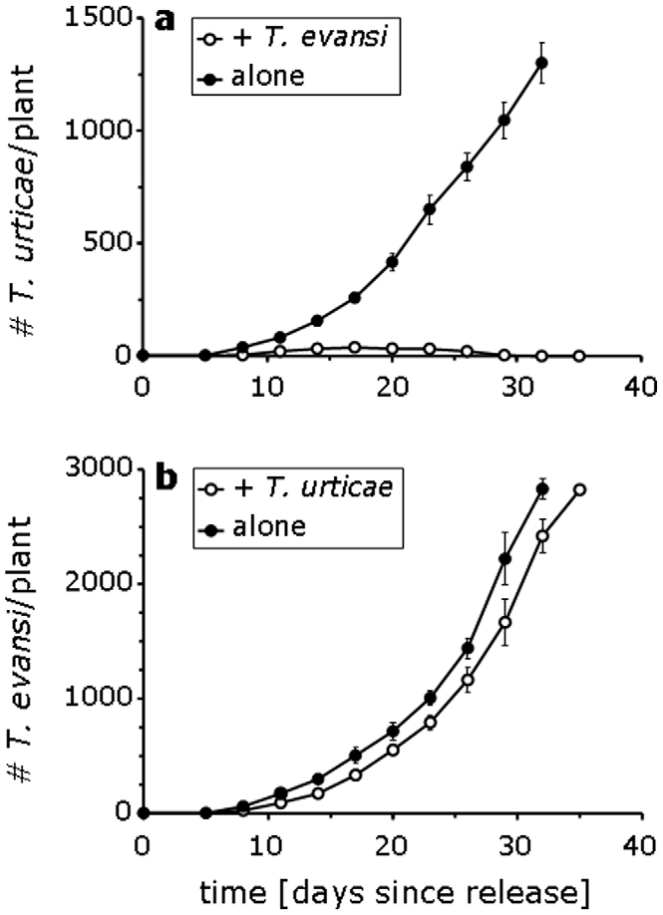
Population dynamics of *T. evansi* and *T. urticae* on tomato plants. **a** Average numbers (± s.e.m., N = 4 plants) of *T. urticae* when alone on a plant (closed circles) or when together with *T. evansi* (open circles). **b** Average numbers of *T. evansi* when alone (closed circles) or when together with *T. urticae* (open cicrles). At the end of the experiment, the plants were overexploited and mites would start dispersing. Data from the two single-species experiments were presented in [16].

Numbers of *T. urticae* were significantly lower on plants where they were together with *T. evansi* than on plants free of *T. evansi* (lme, F_1,6_ = 160.7, p<0.0001, Fig. 2a). The population of *T. urticae* showed a slight increase in the first two weeks, but then declined and went extinct during the last 3 weeks of the experiments. In contrast, there was no significant difference between the population size of *T. evansi* on plants where it was together with *T. urticae* and the plants where it was alone (lme, F_1,6_ = 3.53, p = 0.108, Fig. 2b). The population of *T. evansi* increased over the 5 weeks of the experiments (Fig. 2b), and reached much higher levels than the population of *T. urticae*, even when the latter was alone on a plant (Fig. 2).

### Does web of *T. evansi* hinder *T. urticae*?

**Figure 3:**
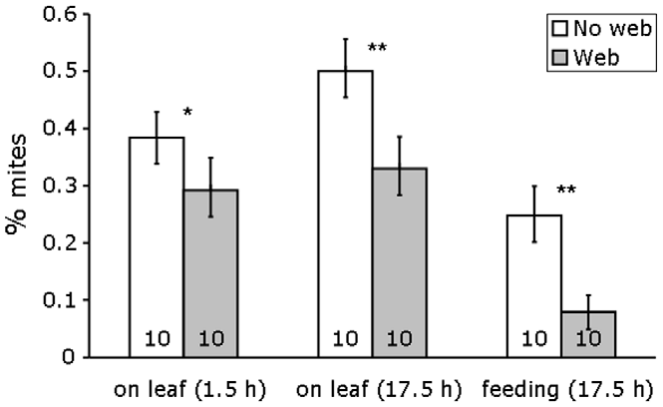
Web of *T. evansi* hinders competitors. The average proportion (+s.e.m., N = 10) of adult female *T. urticae* on the leaf surface after 1.5 h (left-hand two bars), after 17.5 h (middle two bars) and the average proportion of mites feeding after 17.5 h (right-hand two bars), on tomato leaflets that were infested with 100 adult female *T. evansi*. The web produced by *T. evansi* was removed from half of the leaves (web removed), and was left intact on the other half of the leaflets. Asterisks above the bars indicate significant difference between the two treatments: *: p<0.05; **: p<0.005.

Although we tried to carefully remove the web from half of the leaflets, there was invariably some web left, and the mites furthermore continued producing web during the experiment. Therefore, we found mites in the web in both treatments, but fewer in the treatment without web (55% with web vs 17% with web removed after 1.5 h and 57.4% vs 12.5% after 17.5 h). After 1.5 and 17.5 h, a significantly lower proportion of adult females were found on the leaf surface when web was not removed (Fig. 3: glm: 1.5 h: Chi^2^ = 4.42, d.f. = 1, p = 0.035; 17.5 h: Chi^2^ = 10.08, d.f. = 1, p = 0.0015). When most of the web was removed, the proportion of mites on the leaf surface increased over time (Fig. 3). This did not occur when the web was not removed. Significantly more mites were feeding when web was removed than when the web was left intact (Fig. 3: glm: Chi^2^ = 10.10, d.f. = 1, p = 0.0015).

### Webbing and oviposition by *T. evansi*

**Figure 4:**
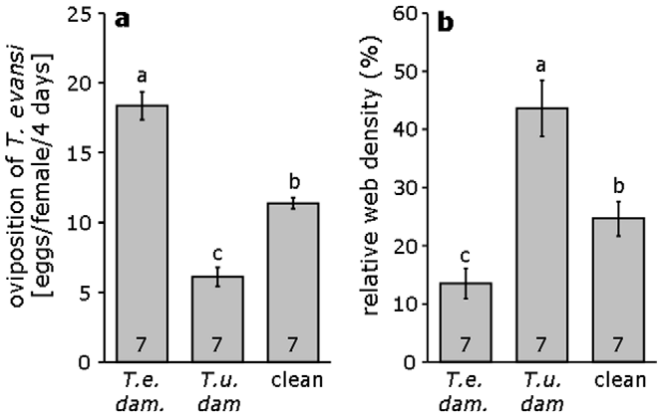
Local cues of competitors induce web production in *T. evansi*. **a** Mean (+s.e.m., N = 7) oviposition rate of *T. evansi* on tomato leaf discs that were previously infested with conspecific mites (T.e. dam.), with *T. urticae* (T.u. dam.), and on clean leaves (clean). **b** Mean (+s.e.m.) relative web density of *T. evansi* on tomato leaf discs that were previously infested with conspecifics (T.e. dam.), with *T. urticae* (T.u. dam.), and on clean leaves (clean). Different letters denote significant differences among treatments (lme with log likelihood ratio test). Numbers inside the bars are number of replicates.

The oviposition of *T. evansi* was significantly affected by previous damage by *T. urticae* or by conspecifics (Fig. 4a, lme: L-ratio = 43.7, d.f. = 2, *p*<0.0001). As reported before [16], the oviposition rate was significantly higher on tomato leaf discs that previously received damage by conspecifics than on leaves that were previously damaged by *T. urticae* or on clean discs (Fig. 4a. L-ratio = 47.2 and 28.1 respectively, *p*'s<0.0001). As found above, the oviposition rate of *T. evansi* was significantly lower on leaf discs that were previously damaged by *T. urticae* than on clean leaf discs (L-ratio = 20.2, *p*<0.0001) (cf. Fig. 2b).

There was a significant effect of treatment on the web production by *T. evansi* (lme: Fig. 4b, L-ratio = 23.5, d.f. = 2, *p*<0.0001). The relative web density was a factor 3 higher on leaf discs from plants that were previously damaged by *T. urticae* than on leaf discs from plants that had received damage by *T. evansi* (L-ratio = 23.4, *p*<0.0001), and almost a factor 2 higher on clean discs than on discs previously damaged by *T. evansi* (L-ratio = 6.38, *p* = 0.012).

### Effect of conspecific and heterospecific cues on oviposition and web production by *T. evansi*

**Figure 5:**
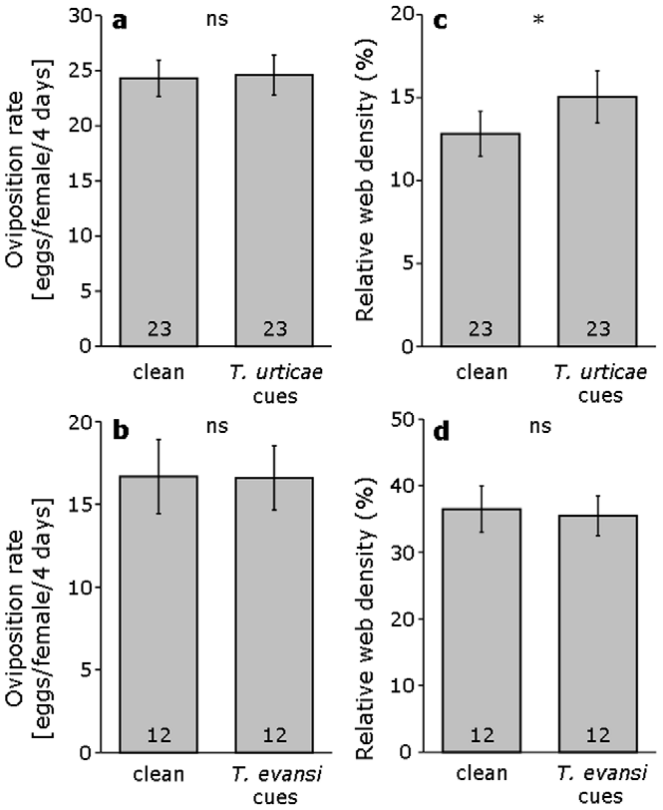
Distant cues of competitors induce web production in *T. evansi*. Effect of cues from clean leaves or leaves infested by *T. urticae* or *T. evansi* on the production of eggs and web by *T. evansi*. **a** Effect of cues from leaves with *T. urticae* on oviposition of *T. evansi*. **b** Effect of cues from leaves with *T. evansi* on oviposition of *T. evansi*. **c** Effect of cues from leaves infested with *T. urticae* on web production of *T. evansi*. **d** Effect of cues from leaves infested with *T. evansi* on web production of *T. evansi*. * denotes a significant difference between treatments (lme with log likelihood ratio test, *p* = 0.03); ns means no significant differences (p>0.05). For logistical reasons, experiments of panels **a** plus **b** and **c** plus **d** were not carried out at the same time. Therefore, treatments can only be compared to their controls within the same experiment. Numbers inside bars give the number of replicates.

The rate of oviposition of *T. evansi* was not affected by cues from leaflets infested with *T. urticae* (lme, L-ratio = 0.05, d.f. = 1, *p* = 0.82, Fig. 5a), or with conspecific mites (lme, L-ratio = 0.006, d.f. = 1, p = 0.94, Fig. 5b). However, there was a significant effect of cues from leaflets infested by *T. urticae* on web production by *T. evansi* (lme, L-ratio = 5.0, d.f. = 1, p = 0.025, Fig. 5c), but not of cues from leaflets infested with conspecifics (lme, L-ratio = 0.15, d.f. = 1, p = 0.7, Fig. 5d).

## Discussion

We reported earlier that the damage caused by *T. evansi* reduces tomato plant defences to such low levels that the plants become of higher quality for this mite species than clean plants [16]. Here, we show that *T. urticae* can profit from this increase in plant quality; it had an almost 2-fold higher oviposition rate on leaf discs from plants on which *T. evansi* had fed previously and from which the web of *T. evansi* was removed. This suggests that from the viewpoint of *T. urticae*, the quality of the leaf tissue increased as a result of the feeding activities by *T. evansi*. *Tetranychus evansi* down-regulates the production of compounds associated with plant defence (such as proteinase inhibitors) within the leaves of its host plants [16], whereas *T. urticae* up-regulates it [14].

Although oviposition rates of *T. urticae* were lower on leaves with damage and web of *T. evansi* than on leaves with damage but without web, it was still significantly higher than on clean plants. This implies that *T. urticae* could benefit from the down-regulated defence despite the hinder it experienced from the *T. evansi* web. In contrast, females of *T. evansi* were apparently not hindered by the web of *T. urticae*, but they had lower oviposition rates on plants damaged by *T. urticae* than on clean plants, suggesting that they were negatively affected by the plant defences induced by *T. urticae*.

These results suggest that *T. evansi* would suffer from competition with *T. urticae*, but that *T. urticae* would actually profit from the presence of *T. evansi* on the same plant. Yet, we found the opposite: *T. evansi* always outcompeted *T. urticae* when present on the same plants. We hypothesize that *T. evansi* uses its profuse web to prevent *T. urticae* from profiting from the decreased plant defence induced by *T. evansi*. Indeed, we show that *T. urticae* had difficulties reaching the leaf surface to feed on plant cells when this surface was covered with web produced by *T. evansi*. In spider mites, feeding, food conversion into egg biomass and oviposition are closely related, whereas the population growth rate is closely related to the rate of oviposition [28]. Adult females feed on plants to produce up to 50–70% of their own weight in eggs per day [36]. Hence, the reduced oviposition rate of *T. urticae* in the presence of web of *T. evansi* was probably caused by *T. urticae* experiencing difficulties in reaching the leaf surface, resulting in reduced feeding. A reduced feeding rate will result in a lower rate of oviposition and a reduced rate of population growth, which may partly explain the competitive displacement of *T. urticae* by *T. evansi*.

The finding that *T. evansi* increased web production when perceiving cues associated with *T. urticae* suggests that the increased web production of *T. evansi* serves to retard the population growth of its competitor. Possibly, *T. evansi* won the competition on individual plants because of this increased web production. The nature of the cues that induce extra web production in *T. evansi* is not yet clear and deserves further research.

It also remains to be investigated how the competition between the two species would end when populations of the two species are not released simultaneously. For example, if *T. urticae* would have a head start, it could potentially induce defences in the plant, which would slow down the population growth of later-arriving *T. evansi*. In contrast, if *T. evansi* would have a head start, it could produce less dense web because it would not perceive cues associated with *T. urticae*, and the latter species might have easier access to the high-quality leaf area occupied by *T. evansi*.

Another question that remains to be answered is why *T. evansi* does not always produce such high amounts of web. It suggests that the flexibility of web production by *T. evansi* is an adaptation to the heterogeneity of the environment and that the increased production of web by *T. evansi* on plants damaged by *T. urticae* may be an induced defence against competition. Thus, there may be a trade-off between silk production and egg production. The silk of the web produced by spider mites consists of proteins [37] and its production may thus go at the expense of amino acids that would otherwise be available for growth and reproduction. However, we did not find indications for such a trade-off; *T. evansi* produced more web in response to cues from leaves with *T. urticae* but did not produce fewer eggs (Fig. 5).

Our results show that the down-regulation of plant defences by *T. evansi* [16] also has positive effects on another, closely related spider mite species. This is a potential ecological cost of such down-regulation, which does not occur when herbivores become resistant to plant defences instead of down-regulating them [11]. The results suggest that *T. evansi* circumvents these costs through the production of profuse web over the leaf area where it has triggered the down-regulation of plant defences, and it is this web that hinders interspecific competitors. Shielding off competitors with a dense web would only function if plant feeding by *T. evansi* results in a local, non-systemic, repression of plant defence, most ideally restricted to the area occupied by its web. Indeed, earlier research indicates that the repression of plant defence by *T. evansi* is a local phenomenon [16]. Thus, it seems that *T. evansi* protects the leaves on which it resides against invasions by competitors through the production of web.

It is striking that *T. evansi* reached much higher levels on tomato plants than did *T. urticae*, even when the latter species was present alone (Fig. 2). The reason for this is the higher population growth rate of *T. evansi* [16], which is caused by the reduction in plant defences. Hence, on the one hand, *T. evansi* reduces plant defences, thus reaching a higher population growth rate, and on the other hand, it prevents heterospecific competitors from obtaining similarly high growth rates by protecting the leaf area with reduced plant defences with web.

A further issue that needs to be addressed is that conspecific competitors can also profit from the down-regulation of plant defences, and the web produced by *T. evansi* obviously cannot exclude these competitors. Although the spider mites inside a colony will mostly be offspring from the same foundress and thus kin-related [38], colonies of *T. evansi* that down-regulate plant defences can, in theory, be invaded by conspecifics that do not invest in down-regulation of defences, but can profit from the local lower plant defences. This issue clearly needs further study.

We conclude that, besides being a defence against predators [24], the silken web produced by *T. evansi* may also serve to reduce competition with heterospecific competitors [23]. The costs of such web production remain to be assessed. Other ecological costs may be that plant defences can only be down-regulated in a limited range of plant species, thus restricting the host plant range of the herbivore [11].

## Supporting Information

Statistical Tables S1 A herbivorous mite down-regulates plant defence and produces web to exclude competitors.
(PDF)
