# Supplementary material for: A Herbivorous Mite Down-Regulates Plant Defence and Produces Web to Exclude Competitors
Source: PLoS One. 2011 Aug 24;6(8):e23757. doi: 10.1371/journal.pone.0023757 (PMC3161068; doi:10.1371/journal.pone.0023757)
Supplement: Statistical Tables S1 — A herbivorous mite down-regulates plant defence and produces web to exclude competitors. (PDF) [file pone.0023757.s001.pdf]

Statistical tables with Sarmento *et al.*

**A herbivorous mite down-regulates plant defence and produces web to exclude competitors**

All analyses were done in R (v. 2.12.0) [1]. Notice that we present the full models here in order to give magnitude of the effects ("Value" in the tables below). The statistics presented in the main text were obtained through comparing models with and without the respective factor with the "anova" function in R [2].

Ad Figure 1a: Linear mixed effects model (LME) of oviposition by *T. urticae* on clean leaf discs and leaf discs damaged by *T. evansi,* either with or without web produced by *T. evansi*.

```
Linear mixed-effects model fit by maximum likelihood
Random effect (Replicate):
        (Intercept) Residual
StdDev:    3.085     4.454

Fixed effects:
                  Value*   S.E.     DF  t value    p
Clean              7.38    1.05    254    7.00   0.000
Damaged           11.04    0.67    254   16.40   0.000
Damaged + web      4.78    0.68    254    7.02   0.000

* Here and in the tables to follow, the first value in this column is the size of
the first fixed effect, subsequent values are relative to this first value (the
value of "Clean" is 7.38, that of "Damaged" is 7.38 + 11.04, that of "Damaged +
web" is 7.38 + 4.78, see Figure 1a).
```

Ad Figure 1b: LME of oviposition by *T. evansi* on clean leaf discs and leaf discs damaged by *T. urticae,* either with or without web produced by *T. urticae*.

```
Linear mixed-effects model fit by maximum likelihood
Random effect (Replicate):
        (Intercept) Residual
StdDev:    1.148     3.591

Fixed effects:
                  Value    S.E.     DF  t value    p
Clean             13.75    1.25     42   11.00   0.000
Damaged           -4.83    1.39     42   -3.48   0.001
Damaged + web     -5.11    1.30     42   -3.94   0.000
```

Ad Figure 2a: LME of numbers of *T. urticae* on plants with or without *T. evansi* (Treatment). Notice that we do not report the significant effect of Time and the significant interaction of Time and Treatment in the manuscript for brevity.

```
Linear mixed-effects model fit by maximum likelihood
Random effect (Replicate):
         (Intercept) Residual
StdDev:    5.02e-05   1.049

Fixed effects:
                  Value    S.E.     DF  t value    p
with T. evansi     2.65    0.32     79    8.36   0.000
w.o. T. evansi    -0.94    0.45      6   -2.08   0.082
Time              -0.03    0.02     79   -2.08   0.041
Treatment:Time     0.22    0.02     79    9.88   0.000
```

Ad Figure 2b: LME of numbers of *T. evansi* on plants with or without *T. urticae* (Treatment).

Linear mixed-effects model fit by maximum likelihood
Random effect (Replicate):

| | (Intercept) | Residual |
|---|---|---|
| StdDev: | 2.95e-05 | 0.709 |

Fixed effects:

| | Value | S.E. | DF | t value | p |
|---|---|---|---|---|---|
| with *T. urticae* | 1.53 | 0.21 | 79 | 7.13 | 0.000 |
| w.o. *T. urticae* | 0.37 | 0.31 | 6 | 1.21 | 0.269 |
| Time | 0.21 | 0.01 | 79 | 19.73 | 0.000 |
| Treatment:Time | 0.00 | 0.03 | 79 | 0.01 | 0.993 |

Ad Figure 3: Generalized Linear Model (GLM with binomial error distribution) of the percentage of *T. urticae* that reached the leaf surface covered with web of *T. evansi* or without web, and the percentage of *T. urticae* that was feeding.

On the leaf surface:

| | Value* | S.E. | z value | p |
|---|---|---|---|---|
| No web (1.5h) | -0.23 | 0.21 | -1.08 | 0.282 |
| Web (1.5h) | -0.65 | 0.31 | -2.09 | 0.037 |
| No web (17.5h) | 0.58 | 0.24 | 2.46 | 0.014 |
| Web (17.5h) | -1.02 | 0.33 | -3.12 | 0.002 |

Feeding:

| | Value* | S.E. | z value | p |
|---|---|---|---|---|
| No web | -0.99 | 0.25 | -3.91 | 9.17e-05 |
| Web | -1.40 | 0.47 | -2.98 | 0.002 |

* Notice that the estimate is on a transformed, logit, scale. For back-transformation to proportion $p = 1/(1+1/e^{x})$, with x being the value.

Ad Figure 4a: LME of oviposition by *T. evansi* on leaf discs damaged by *T. evansi*, by *T. urticae*, or clean.

Linear mixed-effects model fit by maximum likelihood
Random effect (Replicate):

| | (Intercept) | Residual |
|---|---|---|
| StdDev: | 0.967 | 1.513 |

Fixed effects:

| | Value | S.E. | DF | t value | p |
|---|---|---|---|---|---|
| Clean | 11.37 | 0.73 | 12 | 15.51 | 0.000 |
| Damage *T. evansi* | 7.00 | 0.87 | 12 | 8.01 | 0.000 |
| Damage *T. urticae* | -5.26 | 0.87 | 12 | -6.02 | 0.000 |

Ad Figure 4b: LME of web production by *T. evansi* on leaf discs damaged by *T. evansi*, by *T. urticae*, or clean.

Random effect (Replicate):

| | (Intercept) | Residual |
|---|---|---|
| StdDev: | 2.95e-06 | 0.088 |

Fixed effects:

| | Value* | S.E. | DF | t value | p |
|---|---|---|---|---|---|
| Clean | 0.25 | 0.04 | 12 | 6.85 | 0.000 |
| Damage *T. evansi* | -0.11 | 0.05 | 12 | -2.19 | 0.049 |
| Damage *T. urticae* | 0.19 | 0.05 | 1 | 3.73 | 0.003 |

* Value is given as proportion, data in Figure 4b are percentages.

Ad Figure 5a and b: LME of oviposition by *T. evansi* when exposed to distant cues of leaves with *T. urticae* or with *T. evansi,* or exposed to clean leaves.

```
Linear mixed-effects model fit by maximum likelihood
Random effect (Replicate):
        (Intercept) Residual
StdDev:    6.991     4.184

Fixed effects:
                  Value    S.E.     DF  t value    p
Clean             24.31    1.74     22   13.99   0.000
T. urticae         0.28    1.26     22    0.22   0.826

Random effect (Replicate):
        (Intercept) Residual
StdDev:    6.462     2.599

Fixed effects:
                  Value    S.E.     DF  t value    p
Clean             16.68    2.10     11    7.94   0.000
T. evansi         -0.08    1.11     11   -0.07   0.942
```

Ad Figure 5c and d: LME of web production by *T. evansi* when exposed to distant cues of leaves with *T. urticae* or with *T. evansi,* or exposed to clean leaves.

```
Linear mixed-effects model fit by maximum likelihood
Random effect (Replicate):
        (Intercept)   Residual
StdDev:    0.061       0.032

Fixed effects:
                  Value*   S.E.     DF  t value    p
Clean              0.13    0.01     22    8.78   0.000
T. urticae         0.02    0.01     22    2.31   0.031

Random effect (Replicate):
        (Intercept)   Residual
StdDev:    0.088       0.062

Fixed effects:
                  Value*   S.E.     DF  t value    p
Clean              0.37    0.03     11   11.27   0.000
T. evansi         -0.01    0.03     11   -0.37   0.715
```

* Value is given as proportion, data in Figure 5c,d are percentages.

**References**


1. R Development Core Team (2010) R: A language and environment for statistical computing. Vienna, Austria: R Foundation for Statistical Computing.
2. Crawley MJ (2007) The R Book. Chichester, England: John Wiley & Sons Ltd. 942 p.
